# Are Germ Diseases Practically Non-Communicable?

**Published:** 1913-04

**Authors:** 


### Are Germ Diseases Practically Non-Communicable?

Soon after the general acceptance of the "germ theory," there was a natural exaggeration of the importance of contact or even of propinquity in spreading disease. Of late, the pendulum has swung rather to the opposite extreme. Numerous articles have appeared in regard to tuberculosis, and while no author has been so bold as to deny the existence of the tubercle bacillus, there has been a tendency to the pessimistic view that the bacillus was so ubiquitous that any one predisposed to the disease would contract it somehow, some time, and somewhere, while one not so predisposed might expose himself to the implantation of the bacillus with impunity. Perhaps no one has so expressed himself but many have plainly implied such a view, in estimating the practical importance of exposure. Similarly, with regard to measles, and with a more or less explicit extension of the argument to other diseases usually considered actively contagious, it has been stated that the alcove arrangement of wards is sufficient to prevent infection, that the disease is not air-borne, and that disinfection might be omitted or has actually been discontinued without increase in the incidence of the disease.

Without in the least questioning the importance of predisposing and contributing factors in securing the implantation of a specific disease germ—and we say *germ* instead of bacterium, because a great many such organisms are protozoa, fungi, etc.—we feel that the essential element is, after all, the germ itself. Almost all disease germs are nearly absolutely obligate parasites, that is to say, they would succumb to ordinary vicissitudes of life outside of a host, in a comparatively short time, though also capable of retaining infective virulence for long periods under favorable conditions. In the case of a germ which is infectious only in a definite stage of its life cycle, or which requires some very special mode of implantation, contagiousness may be reduced to a theoretic degree. Otherwise, we believe that no chances should be taken and that every known method of preventing infection should be practiced.

